# Diabetic Patients’ Awareness and Knowledge of the Association Between Apical Periodontitis and Diabetes Mellitus: A Cross-Sectional Study

**DOI:** 10.3390/healthcare14213619

**Published:** 2026-10-26

**Authors:** Sevinç Aktemur Türker, Maide Nazlı Kaya, Nazife Maide Dayıcan, Taner Bayraktaroğlu

**Affiliations:** 1 Department of Endodontics, Faculty of Dentistry, Zonguldak Bülent Ecevit University, Zonguldak 67000, Turkey; 2 Faculty of Dentistry, Zonguldak Bülent Ecevit University, Zonguldak 67000, Turkey; 3 Department of Endocrinology and Metabolism, Faculty of Medicine, Zonguldak Bülent Ecevit University, Zonguldak 67000, Turkey

**Keywords:** periapical periodontitis, diabetes mellitus, patient awareness, health knowledge, attitude, survey

## Abstract

**Highlights:**

**What are the main findings?**

- Diabetic patients showed knowledge gaps about the link with apical periodontitis.
- Education was the only factor associated with both knowledge and awareness.
- This association remained significant after multivariable adjustment.

**What are the implications of the main findings?**

- Targeted oral health education may benefit diabetic patients.
- Patients with lower education levels may benefit most.
- Integrating oral health education into diabetes care may improve disease awareness.
- It may also promote earlier dental attendance.

**Abstract:**

**Backgrounds/Objectives:** The objective of this study was to evaluate diabetic patients’ knowledge and awareness regarding the association between diabetes mellitus and apical periodontitis and to examine demographic and clinical factors associated with knowledge and awareness scores. **Methods:** A cross-sectional study was conducted involving 504 patients with diabetes who attended the Obesity and Diabetes Research and Application Center between September 2025 and February 2026. Data regarding demographic characteristics, clinical status, and knowledge/awareness of the DM-apical periodontitis link were collected via a structured questionnaire. Statistical analyses were performed using the Kruskal–Wallis test followed by Dunn’s multiple comparison test, the Mann–Whitney U test, and Spearman’s correlation analysis (*p* < 0.05). Separate multiple linear regression models were also used to identify factors independently associated with knowledge and awareness scores. **Results:** The majority of participants were aged 51–80 (76.0%), female (59.1%) and had completed primary education (41.5%, *n* = 209). While 84% acknowledged the need for regular dental check-ups, 49.1% visited a dentist only for emergencies, and 85.9% lacked prior knowledge of apical periodontitis. Overall, 62.7% of participants recognized the importance of glycemic control for the healing of apical periodontitis, and 52.8% identified diabetes as a risk factor for apical periodontitis. A notable knowledge gap was observed regarding the reverse relationship: only 40.5% recognized that apical periodontitis may adversely affect diabetes control, whereas 53.0% reported having no idea. The mean knowledge score was 2.65 ± 1.97, and the mean awareness score was 26.57 ± 3.21. A strong positive correlation was found between knowledge and awareness scores (*r* = 0.605, *p* < 0.001). Knowledge and awareness scores differed significantly according to age and education level (*p* < 0.01 and *p* < 0.001, respectively). Knowledge and awareness scores also differed significantly according to dental-visit frequency and previous tooth-extraction history (*p* < 0.001). Gender and diabetes duration were not significantly associated with either knowledge or awareness scores (*p* > 0.05). In the multivariable analyses, education was the only factor that remained significantly associated with both knowledge and awareness scores after adjustment for the other variables included in the models. **Conclusions:** Important gaps were identified in diabetic patients’ knowledge and awareness regarding the association between apical periodontitis and diabetes mellitus, particularly concerning the potential relationship between apical periodontitis and glycemic control. Future studies should evaluate whether targeted educational interventions can improve endodontic-related health literacy and preventive dental-care behaviors.

## 1. Introduction

Apical periodontitis is an inflammatory infectious disease of polymicrobial origin. It is characterized by the destruction of the periodontal ligament and resorption of the alveolar bone. This occurs when microorganisms, toxins, enzymes, and metabolic products within the root canal spread outside the root. It usually results from severe caries, pulp lesions, or periodontal diseases. According to a systematic review and meta-analysis, 52% of the global adult population has at least one case of apical periodontitis [1]. Research reports that untreated infections can lead to tooth loss, jawbone inflammation, and even deaths related to systemic diseases [2,3,4]. Studies have shown that apical periodontitis modulates the systemic immune response by changing the levels of inflammatory cytokines such as C-reactive protein, tumor necrosis factor-α, IL-6, and IL-1. This indicates that inflammation of the root canal plays an important role in overall health [5,6,7]. Furthermore, the long-term persistence of a chronic inflammatory disease has been reported to alter circulating inflammatory factors and cause systemic immune dysfunction [8].

The relationship between apical periodontitis and the host not only creates challenges for root canal treatment; it also acts as a potential risk factor for systemic diseases in other parts of the body [9]. Recent studies have demonstrated a bidirectional interaction between apical periodontitis and various systemic diseases [9,10,11]. Diabetes Mellitus (DM) is one of these chronic systemic diseases that shares such a relationship. Diabetes Mellitus is one of the most common chronic metabolic disorders which is characterized by disruptions in carbohydrate, fat, and protein metabolism due to defects in insulin secretion or its mechanism of action. The disease is marked by hyperglycemia, which directly affects the integrity of the dental pulp, causes difficulties in wound healing, and leads to both systemic and oral symptoms. As one of the most prevalent metabolic diseases, the global prevalence of DM for all age groups is expected to rise from 2.8% in 2000 to 4.4% by 2030, potentially affecting 693 million people or more by 2045 [12,13].

The literature contains numerous studies evaluating apical periodontitis in diabetic patients [14,15,16,17,18,19,20]. It has been reported that the presence of Diabetes Mellitus leads to an increased incidence of root canal infections, delayed healing of apical periodontitis, and a higher prevalence of extractions in teeth with prior root canal treatment [15]. A systematic review reported that oral complications in diabetic patients, such as root canal infections, affect the patients’ quality of life [21]. Especially in patients with uncontrolled diabetes, a poor prognosis is observed, with tooth loss occurring due to delayed or impaired healing in the periapical region. It has been noted that as the severity of DM increases, alveolar bone loss accelerates and the inflammatory response in apical tissues intensifies [22].

Many studies in the literature have investigated the potential link between DM and apical periodontitis [15,23,24,25]. In this bidirectional relationship, DM increases the prevalence of apical periodontitis, accelerates its progression, and weakens the effectiveness of root canal treatment. Conversely, apical periodontitis has been reported to affect insulin signaling pathways or reduce insulin sensitivity, leading to an increased need for insulin and higher blood sugar levels. A recent prospective cohort study reported that the presence of Type 2 DM delays the healing of apical periodontitis. Conversely, the presence of apical periodontitis contributes to an increased inflammatory burden in Type 2 DM. It has been noted that performing root canal treatment reduces the cumulative inflammatory burden in Type 2 DM, thereby contributing to improved glycemic control, especially in patients with HbA1c ≥7% [26]. Given the prevalence of misinformation and the lack of awareness among the general population, many individuals are unable to self-screen for both conditions, which can have a significant impact on the prognosis for both diseases. Therefore, to successfully manage, treat, and prevent both apical periodontitis and DM, it is essential to increase patients’ awareness and knowledge levels. The objective of this study was to evaluate diabetic patients’ knowledge and awareness regarding the association between diabetes mellitus and apical periodontitis and to examine demographic and clinical factors associated with knowledge and awareness scores.

## 2. Materials and Methods

### 2.1. Study Design and Ethical Approval

This cross-sectional survey-based study was conducted between September 2025 and February 2026. The study protocol was reviewed and approved by the Non-Interventional Clinical Research Ethics Committee of the University (Protocol No: 2025/02). The study was conducted in accordance with the principles of the Declaration of Helsinki. All participants were informed about the aim of the study, and written informed consent was obtained prior to participation. The reporting of this study was guided by the Strengthening the Reporting of Observational Studies in Epidemiology (STROBE) guidelines for cross-sectional studies [27].

### 2.2. Study Population and Data Collection

A total of 504 patients diagnosed with diabetes mellitus (Type 1 or Type 2) who applied to the Obesity and Diabetes Research and Application Center of the University were included in the study. Eligible patients attending the center during the study period were consecutively invited to participate. The inclusion criteria were being aged 18 years or older; having a confirmed diagnosis of DM; and being willing to participate in the study. Patients with cognitive impairments or those who declined to participate were excluded.

The questionnaire was adapted from extant literature investigating oral health awareness in diabetic populations and was subsequently tailored to meet the specific objectives of the current study. The target sample size was determined pragmatically based on feasibility and the expected number of eligible patients attending the study center during the predefined recruitment period, and the aim of obtaining a sufficiently large sample to allow stable estimation of knowledge and awareness scores. Approximately 500 participants were targeted, and 504 participants were ultimately included. Data collection followed a hybrid approach to maximize the response rate and ensure a representative sample. While the primary data collection tool was a digital questionnaire (Google Forms, (Google LLC, Mountain View, CA, USA), physical paper-based versions of the same questionnaire were provided to participants who preferred traditional completion or had no digital devices. To maintain data consistency, all responses from the paper forms were manually entered into the digital database by two independent researchers and cross-checked for accuracy.

### 2.3. Survey Instrument and Content

The survey instrument was adapted from validated studies in the literature [28,29,30,31] and modified to align with the specific objectives of the current study (Questionnaire S1: Diabetic patients’ knowledge and awareness of the relationship between diabetes mellitus and apical periodontitis). The survey consisted of 27 questions structured into three distinct sections as follows:

Demographic and Clinical Characteristics: Consisted of 14 questions covering age, gender, type and duration of diabetes, glycemic control parameters, educational level, and oral hygiene habits, etc.

Knowledge Assessment: Included 5 “Yes/No/No Idea” questions regarding the link between diabetes and root canal infections. For the knowledge scale, correct responses were scored as 1, whereas incorrect and “No Idea” responses were scored as 0. The scores of the five items were summed to obtain a total knowledge score ranging from 0 to 5, with higher scores indicating greater knowledge.

Awareness Assessment: Comprised 8 statements evaluated using a 5-point Likert scale (1 = strongly disagree, 2 = disagree, 3 = neutral, 4 = agree, and 5 = strongly agree) regarding the awareness of individuals about the bidirectional relationship between diabetes mellitus and apical periodontitis.

Content validity was assessed by two endodontists and one endocrinologist to ensure clinical relevance. Subsequently, a pilot study was conducted with a representative group of 20 diabetic patients (not included in the final analysis) to evaluate the clarity and comprehensibility of the items. Following the main data collection from 504 participants, the structural validity and reliability of the questionnaire were rigorously evaluated. The structural validity and reliability of the questionnaire were evaluated using Exploratory Factor Analysis (EFA) and Confirmatory Factor Analysis (CFA). For structural validation, the total sample (*n* = 504) was randomly divided into two independent subsamples. Exploratory factor analysis (EFA) was performed in the first subsample (*n* = 252), and confirmatory factor analysis (CFA) was subsequently conducted in the second independent subsample (*n* = 252) to evaluate the factor structure identified by EFA.

For the Knowledge Scale, EFA revealed a single-factor structure explaining 34.80% of the total variance. The suitability of the data was confirmed by a Kaiser-Meyer-Olkin (KMO) value of 0.829 and a significant Bartlett’s test (*p* < 0.001). CFA results showed an excellent model fit with the following indices: GFI = 0.996, TLI = 0.995, CFI = 0.998, and RMSEA = 0.039.

For the Awareness Scale, EFA identified a two-factor structure explaining 70.92% of the total variance. Item 5 was excluded from the analysis due to cross-loading. The KMO value was 0.809, and Bartlett’s test was significant (*p* < 0.001). Since the data showed a non-normal distribution, the Asymptotically Distribution-Free (ADF) method was used for CFA. After minor modifications, the model demonstrated acceptable fit indices: GFI = 0.932, CMIN/DF = 2.608, and RMSEA = 0.080. All items in both scales exhibited significant factor loadings (*p* < 0.001).

Finally, the internal consistency was verified with Cronbach’s alpha coefficients. Both scales demonstrated high internal consistency, with Cronbach’s alpha values of 0.856 for the knowledge scale and 0.836 for the awareness scale.

Demographic and clinical information, including diabetes type and duration, fasting blood glucose level, medication use, diabetes-related complications, oral health problems, and dental-care behaviors, was self-reported by the participants through the questionnaire. Age, duration of diabetes, and fasting blood glucose levels were analyzed using predefined categorical groups as presented in Table 1.

### 2.4. Statistical Analysis

The statistical analysis was conducted using R software (version 4.1.1; R Foundation for Statistical Computing, Vienna, Austria), IBM SPSS Amos (version 24.0; IBM Corp., Armonk, NY, USA), and JASP (version 0.19.3.0; JASP Team, Amsterdam, The Netherlands). The normality of the data distribution was assessed using the Kolmogorov–Smirnov and Shapiro–Wilk tests.

For group comparisons, the Mann–Whitney U test was used for two-group comparisons, while the Kruskal–Wallis H test was applied for independent variables with three or more categories, followed by Dunn’s post hoc test for multiple comparisons. Relationships between non-normally distributed continuous variables were analyzed using Spearman’s rank correlation coefficient. Quantitative data were presented as mean ± standard deviation and median (minimum: maximum), while categorical data were expressed as frequencies and percentages (*n*, %).

To identify factors independently associated with knowledge and awareness scores, separate multiple linear regression models were constructed for each outcome. Covariates were selected based on clinical and theoretical relevance, together with evidence from previous studies suggesting potential associations with oral health knowledge, awareness, and dental-care behaviors in diabetic populations. Accordingly, age, sex, duration of diabetes, education level, dental-visit frequency, prior knowledge of apical periodontitis and its symptoms, and previous tooth extraction due to a non-healing apical infection were included in the multivariable regression models. Toothbrushing frequency was initially considered for inclusion but was excluded because its variance inflation factor (VIF) exceeded 10, indicating problematic multicollinearity. The retained covariates were entered simultaneously into the models. Regression diagnostics included assessment of residual independence using the Durbin–Watson statistic and multicollinearity using variance inflation factors (VIFs). Durbin–Watson values were 1.752 for the awareness model and 1.780 for the knowledge model, indicating no important residual autocorrelation. Adjusted unstandardized regression coefficients (B) with 95% confidence intervals (CIs) and *p*-values were reported. A *p*-value of <0.05 was considered statistically significant.

## 3. Results

Demographic, clinical, and oral health characteristics are presented in Table 1. Quantitative variables are summarized as mean ± standard deviation and median (minimum–maximum), while categorical variables are reported as frequencies and percentages. Missing responses were observed for a small number of descriptive variables; therefore, analyses were performed using available data for each variable, and no imputation was performed.

Participants’ responses to the knowledge items are presented in Table 2, while responses to the awareness items are summarized in Table 3. The greatest uncertainty in the knowledge assessment concerned the potential adverse effect of apical periodontitis on diabetes control.

Overall, participants showed relatively high awareness of general oral health and diabetes-related preventive messages, whereas uncertainty was more evident for items addressing the specific endodontic–systemic relationship.

Descriptive statistics, internal consistency coefficients, and the correlation between knowledge and awareness scores are presented in Table 4. Knowledge and awareness scores were positively correlated.

Comparisons of knowledge scores according to demographic and clinical characteristics are presented in Table 5. Knowledge scores differed significantly by age, education level, dental-visit frequency, toothbrushing frequency, prior knowledge of apical periodontitis and its symptoms, and previous tooth extraction due to a non-healing apical infection. No significant differences were observed according to sex or duration of diabetes.

Comparisons of awareness scores according to demographic and clinical characteristics are presented in Table 6. Following the factor analyses, Item 5 was excluded because of cross-loading. The final awareness score was therefore calculated by summing the seven retained items (Items 1, 2, 3, 4, 6, 7, and 8), yielding a possible total score ranging from 7 to 35, with higher scores indicating greater awareness. Awareness scores differed significantly by age, education level, dental-visit frequency, toothbrushing frequency, prior knowledge of apical periodontitis and its symptoms, and previous tooth extraction due to a non-healing apical infection. No significant differences were observed according to sex or duration of diabetes.

The results of the multiple linear regression analyses are presented in Table 7 and Table 8. In the multivariable analyses, education level remained significantly associated with both awareness and knowledge scores after adjustment for the other variables included in the models. For awareness, participants with primary education had lower scores than those with higher education (B = −2.630, 95% CI: −4.903 to −0.357; *p* = 0.024), as did literate participants without formal schooling (B = −5.304, 95% CI: −8.836 to −1.771; *p* = 0.004). Similarly, for knowledge, participants with primary education (B = −1.962, 95% CI: −3.073 to −0.850; *p* = 0.001) and literate participants without formal schooling (B = −1.951, 95% CI: −3.679 to −0.223; *p* = 0.027) had significantly lower scores than those with higher education. None of the other variables included in the models showed statistically significant adjusted associations with either score.

## 4. Discussion

Growing literature points toward a bidirectional association between Diabetes Mellitus and apical periodontitis. While diabetes is known to elevate the prevalence of AP and often compromise the outcome of root canal therapy, AP has been associated with impaired glycemic control, potentially through inflammatory pathways related to insulin resistance and metabolic dysregulation [15,23,24,25,32,33,34]. Given this potential oral–systemic association, ensuring that diabetic patients are well-educated about root canal infections is vital for managing both conditions effectively. However, while patient awareness regarding general oral hygiene is well-documented, an important knowledge gap remains regarding endodontic infections. To our knowledge, this cross-sectional study is the first to directly map out diabetic patients’ knowledge and awareness regarding the specific link between AP and DM. Our findings indicate limited awareness concerning how these deep-seated dental infections interfere with metabolic and systemic health.

Because prior endodontic-specific surveys are limited, direct baseline comparisons are not feasible. Nonetheless, looking at the broader literature on diabetes and periodontal disease awareness offers valuable context. For instance, a well-known study on dental outpatients in India reported that an overwhelming 70% of participants had no idea diabetes was linked to periodontal disease [28]. When we contrast those general trends with our data, the knowledge deficit in endodontics appears substantial. Within our cohort, 85.9% of patients lacked any prior knowledge of AP or its clinical symptoms. This finding is consistent with broader evidence indicating limited oral health knowledge and suboptimal dental-care practices among people with diabetes [35]. Even though more than half of the respondents (52.8%) correctly identified diabetes as a risk factor for developing root canal infections, a major segment (59.5%) did not recognize the reverse relationship—that an untreated periapical lesion may be associated with difficulties in blood sugar control. This knowledge gap in comprehensive understanding mirrors recent insights from a Malaysian study on Type 1 diabetic patients [29]. Those researchers observed a discrepancy where patients with uncontrolled HbA1c levels and active gingival bleeding still perceived their oral health as good, reflecting limited understanding of actual disease control. We captured a similar pattern here, while 62.7% of our participants agreed that proper glycemic control is essential for dental healing, nearly half (49.1%) still maintained a predominantly reactive approach, visiting a dentist only for emergencies. This finding is consistent with recent evidence showing that, among patients with diabetes, higher health literacy and educational attainment are positively associated with preventive dental visits [36,37,38].

Our data also indicated that higher education levels were associated with better knowledge and awareness scores. Similar findings have been reported in diabetic populations, with higher educational attainment and greater health literacy being associated with better oral health awareness and more frequent preventive dental visits [37]. Importantly, education was the only factor that remained significantly associated with both knowledge and awareness scores in the multivariable models. Compared with participants with higher education, those with primary education and those who were literate without formal schooling had significantly lower adjusted scores for both outcomes. This suggests that education may represent a particularly important correlate of endodontic-related health literacy in this population. From a clinical standpoint, this overall gap may be understandable. While periodontal disease eventually presents with unmistakable, patient-reported symptoms like bleeding or loose teeth, AP is typically a silent, asymptomatic intraosseous infection. Consequently, if diabetic patients already struggle to link visible oral problems to their systemic condition, they may be less likely to recognize a potential relationship between a painless, chronic endodontic infection and glycemic control without explicit professional guidance.

The demographic makeup of our study group primarily centered on older adults aged 51–80 (76%), the vast majority of whom were managing Type 2 Diabetes (94.8%). This specific window is clinically significant because it captures the stage of life where the cumulative, long-term damage of chronic hyperglycemia becomes most evident. Notably, 57.4% of these participants presented with fasting blood sugar levels exceeding 126 mg/dL, suggesting suboptimal glycemic control in a substantial proportion of the sample. While our primary focus was evaluating patient awareness, this high rate of elevated fasting glucose is clinically relevant. Uncontrolled glucose levels have been associated with a systemic environment that intensifies periapical inflammation and delays healing after endodontic interventions [39]. These findings support the potential value of closer communication between physicians and dentists in the care of this patient population.

Another finding was that despite widespread oral complaints like dry mouth (66.3%) and dental caries (52.8%), 49.1% of patients still only sought dental care during a crisis. This pain-driven behavior may be particularly concerning for diabetic individuals, as AP can remain entirely quiet and asymptomatic while potentially contributing to systemic inflammatory burden. This widespread lack of endodontic literacy (85.9%) may represent an important barrier to preventive dental care and may partly contribute to the relatively low proportion of participants reporting 6-month dental visits. Without realizing that a hidden root canal infection may be associated with poorer glycemic regulation, patients remain trapped in a cyclical pattern of emergency-only treatment. These trends suggest that endodontic health may warrant greater attention within multidisciplinary diabetes care, supporting a more integrated approach to patient education.

A strong positive correlation was observed between knowledge and awareness scores (*r* = 0.605, *p* < 0.001), indicating that greater knowledge was associated with greater awareness. This finding suggests that although participants appeared to recognize general oral–systemic health messages, their understanding of the specific relationship between apical periodontitis and glycemic control remained limited. However, a notable difference was observed in what patients actually understand. While 53.8% of participants agreed or strongly agreed that high oral bacterial loads in diabetic patients could make blood glucose regulation more difficult, only 40.5% recognized the specific potential adverse relationship between apical periodontitis and diabetes control. This suggests that general oral–systemic health messages may be better recognized than the specific role of chronic endodontic infections. In the unadjusted analyses, younger participants demonstrated higher knowledge and awareness scores; however, age was no longer significantly associated with either outcome after multivariable adjustment. Similarly, previous tooth-extraction history was associated with higher scores in the unadjusted comparisons, but this association did not remain statistically significant in the adjusted models. Diabetes duration was not significantly associated with either score in either the unadjusted or adjusted analyses. These findings indicate that some of the associations observed in the unadjusted comparisons may have been influenced by educational level or other correlated characteristics. Collectively, these findings support the potential value of multidisciplinary education that includes endodontic health within broader oral–systemic health education.

From a clinical perspective, the observed gaps in knowledge regarding the bidirectional relationship between diabetes mellitus and apical periodontitis suggest that oral health education for diabetic patients may benefit from greater emphasis on endodontic infections and their potential systemic relevance. Closer communication between dental and medical professionals may also help reinforce consistent oral health messages in this population. From a research perspective, multicenter studies with more diverse diabetic populations are needed to confirm these findings and to determine whether the adjusted associations observed in the present study are reproducible across different populations.

Several limitations should be considered when interpreting the findings of this study. First, the cross-sectional design precludes any causal inference regarding the relationships between demographic or clinical characteristics and knowledge or awareness levels. Second, because the study was conducted at a single clinical center, the findings may not be fully generalizable to all diabetic populations. Third, the use of self-reported questionnaire data may have introduced recall and response bias, and some participants may have overestimated or underestimated their oral health behaviors or previous clinical experiences. Although consecutive recruitment was used to reduce potential selection bias, the inclusion of patients attending a specialized obesity and diabetes center may still limit the representativeness of the sample. In addition, residual confounding cannot be excluded because not all potentially relevant demographic, behavioral, and clinical factors may have been captured or fully accounted for in the analyses. Although the questionnaire underwent content validation, pilot testing, and psychometric evaluation, its adaptation for the present study and the exclusion of one awareness item during factor analysis should also be considered when interpreting the score-based findings. The absence of an a priori sample-size calculation should also be considered when interpreting the findings, as the study may have had limited ability to detect small associations and the precision of some estimates may be lower than would be ensured by a prespecified power-based sample-size determination. Nevertheless, the study provides a useful baseline assessment of diabetic patients’ knowledge and awareness regarding the relationship between apical periodontitis and diabetes mellitus and may inform future multicenter and longitudinal investigations.

## 5. Conclusions

In conclusion, this study identified important gaps in diabetic patients’ knowledge and awareness regarding the association between apical periodontitis and diabetes mellitus, particularly concerning the potential influence of apical periodontitis on glycemic control. Although knowledge and awareness scores differed according to several demographic and dental-care-related characteristics in the unadjusted analyses, education was the only factor that remained significantly associated with both outcomes after multivariable adjustment. These findings highlight the need for further research to better understand factors associated with endodontic-related health literacy in diabetic populations. Future multicenter studies should also evaluate whether targeted educational interventions can improve patients’ knowledge, awareness, and preventive dental-care behaviors.

## Figures and Tables

**Table 1:** Demographic, clinical, and oral health characteristics of the participants.

Characteristic	Category	*n*	%
Age (years)	18–30	15	3.0
	31–50	94	18.7
	51–80	383	76.0
	>80	12	2.4
Sex	Female	298	59.1
	Male	206	40.9
Diabetes mellitus type	Type 1	26	5.2
	Type 2	478	94.8
Duration of diabetes (years)	1–5	130	25.9
	6–10	130	25.9
	11–15	126	25.1
	>16	116	23.1
Fasting blood glucose (mg/dL)	70–100	34	6.8
	100–125	180	35.9
	>126	288	57.4
Diabetes treatment	Oral antidiabetic drugs	280	55.7
	Oral antidiabetic drugs + insulin	157	31.2
	Insulin only	43	8.6
	No medication	23	4.6
Education level	Illiterate	18	3.6
	Literate without formal schooling	39	7.7
	Primary education	209	41.5
	Secondary education	166	32.9
	Higher education	72	14.3
Occupation	Housewife	235	46.6
	Retired	170	33.7
	Worker/civil servant	55	10.9
	Self-employed	44	8.7
Smoking status	Never smoked	226	44.8
	Former smoker	148	29.4
	Current smoker	130	25.8
Toothbrushing frequency	Never	13	2.6
	Once daily	223	44.3
	Twice daily	212	42.1
	More than twice daily	56	11.1
Dental-visit frequency	Every 2 months	2	0.4
	Every 6 months	143	28.4
	Once a year	110	21.9
	Once every 2 years	1	0.2
	Only for emergencies/when necessary	247	49.1
Prior knowledge of apical periodontitis and its symptoms	Yes	71	14.1
	No	433	85.9
Previous tooth extraction due to a non-healing apical infection	Yes	150	29.8
	No	123	24.4
	No idea	231	45.8
Current oral health problems *	Dry mouth	334	66.3
	Dental caries	266	52.8
	Gingival bleeding during brushing	223	44.2
	Oral ulcers	144	28.6
	Delayed oral wound healing	86	17.1
	No oral health problems	39	7.7
Diabetes-related complications *	Diabetic foot	248	66.0
	Retinopathy	167	44.4
	Nephropathy	53	14.1
	Neuropathy	34	9.0
	Coronary artery disease	26	6.9
	Cerebrovascular disease	14	3.7
	No complications	47	12.5

* Multiple responses were permitted; therefore, percentages may exceed 100%. Percentages were calculated using the available responses for each variable.

**Table 2:** Participants’ responses to knowledge items regarding apical periodontitis.

Knowledge Items	Frequency	Percentage (%)
Diabetes Mellitus increases the risk of apical periodontitis		
Yes	266	52.8
No	14	2.8
No idea	224	44.4
Apical periodontitis follows a more severe course in diabetic patients		
Yes	265	52.6
No	19	3.7
No idea	220	43.7
Diabetes Mellitus makes it harder to treat apical periodontitis		
Yes	286	56.8
No	20	4.0
No idea	198	39.3
The presence of apical periodontitis can prevent Diabetes Mellitus from being controlled		
Yes	204	40.5
No	33	6.5
No idea	267	53
The control of your Diabetes Mellitus is important for the healing of a tooth with apical periodontitis		
Yes	316	62.7
No	11	2.2
No idea	177	35.1

**Table 3:** Distribution of participants’ responses to awareness items regarding apical periodontitis [*n* (%)].

Awareness Items	Strongly Disagree	Disagree	Neutral	Agree	Strongly Agree
In patients with diabetes, apical periodontitis can be more severe or more difficult to manage	-	17 (3.4)	178 (35.3)	278 (55.2)	31 (6.2)
Gingival bleeding and tooth sensitivity require urgent dental treatment, especially for individuals with diabetes	1 (0.2)	28 (5.6)	107 (21.2)	318 (63.1)	50 (9.9)
Diabetic patients are more susceptible to dental infections, including apical periodontitis, than non-diabetic individuals	-	20 (4)	118 (23.4)	315 (62.5)	51 (10.1)
Individuals with diabetes should pay closer attention to their oral health and have regular dental check-ups	-	5 (1)	76 (15.1)	344 (68.3)	79 (15.7)
It is important to inform your dentist about your diabetes and the medications you use to ensure more effective dental treatment	-	7 (1.4)	61 (12.1)	343 (68.1)	93 (18.5)
Lifestyle, diet, and exercise modifications can help manage diabetes, thereby reducing the risk of oral infections such as apical periodontitis	-	10 (2)	132 (26.2)	318 (63.1)	44 (8.7)
High bacterial loads in the mouth of diabetic patients can make it more challenging to regulate blood sugar levels	1 (0.2)	20 (4.0)	212 (42.1)	248 (49.2)	23 (4.6)
Certain diabetes medications may have side effects that affect oral health, such as dry mouth or delayed healing	1 (0.2)	10 (2)	122 (24.2)	320 (63.5)	51 (10.1)

**Table 4:** Descriptive statistics, internal consistency coefficients, and inter-correlations among scales.

	Mean ± Standard Deviation	Median (Minimum–Maximum)	Cronbach’s Alpha		
Knowledge score	2.65 ± 1.97	3 (0–5)	0.856	*r*	*p*
Awareness score	26.57 ± 3.21	27 (14–35)	0.836	0.605	<0.001

*r*: Spearman’s rho correlation. Correlation analysis was performed between Knowledge and Awareness scores, *r*: 0.605, *p* < 0.001.

**Table 5:** Comparison of knowledge scores according to demographic characteristics.

	Mean ± SD	Median (Minimum–Maximum)/Mean Rank	Test Statistic	*p*	ES [95% CI]
Age					
>80	2.75 ± 2.3	3 (0–5)/264.04 ^ab^	14.367	0.002 ^x^	0.023 [−0.0020–0.055]
18–30	3.87 ± 1.64	4 (0–5)/336.2 ^ab^			
31–50	3.18 ± 1.71	4 (0–5)/288.87 ^a^			
51–80	2.47 ± 2	3 (0–5)/239.93 ^b^			
Sex					
Male	2.73 ± 1.98	3 (0–5)/259.05	−0.840	0.401 ^y^	0.075 [−0.103–0.253]
Female	2.6 ± 1.96	3 (0–5)/247.97			
Duration of Diabetes (years)			3.004	0.391 ^x^	0.000 [−0.006–0.006]
>16	2.51 ± 1.99	3 (0–5)/241.01			
1–5	2.62 ± 1.96	3 (0–5)/248.9			
11–15	2.56 ± 1.99	3 (0–5)/245.3			
6–10	2.88 ± 1.94	3 (0–5)/269.47			
Education Level					
Primary	2.01 ± 2.01	1 (0–5)/207.66 ^a^	60.767	<0.001 ^x^	0.114 [0.055–0.176]
Illiterate	1.39 ± 1.72	0.5 (0–5)/160.42 ^a^			
Literate without formal schooling	2.44 ± 1.89	2 (0–5)/237.01 ^ab^			
Secondary	3.16 ± 1.77	3 (0–5)/289.17 ^bc^			
Higher education	3.76 ± 1.52	4 (0–5)/329.54 ^c^			
How often do you visit a dentist?			46.430	<0.001 ^x^	0.087 [0.038–0.144]
Every 2 months	0.5 ± 0.71	0.5 (0–1)/107.75 ^ab^			
Every 6 months	3.36 ± 1.71	4 (0–5)/301.69 ^a^			
Only for emergencies/when necessary	2.06 ± 1.94	2 (0–5)/209.87 ^b^			
Once a year	3.08 ± 1.95	4 (0–5)/282.35 ^a^			
How often do you brush your teeth?			29.734	<0.001 ^x^	0.054 [0.015–0.099]
Once a day	2.23 ± 2.03	2 (0–5)/223.78 ^ab^			
More than twice a day	2.93 ± 1.66	3 (0–5)/267.58 ^ac^			
Twice a day	3.12 ± 1.88	4 (0–5)/285.77 ^c^			
Never	1.08 ± 1.38	0 (0–4)/137.5 ^b^			
Do you know about root canal infections and their symptoms?					
Yes	4.03 ± 1.4	5 (0–5)/352.77	−6.259	<0.001 ^y^	0.581 [0.327–0.834]
No	2.43 ± 1.96	3 (0–5)/236.06			
Have you ever had a tooth extracted due to a non-healing infection at the root canal?					
Yes	2.8 ± 1.86	3 (0–5)/261.97 ^a^	18.696	<0.001 ^x^	0.033 [0.006–0.072]
No idea	1.99 ± 1.94	1 (0–5)/204.78 ^b^			
No	2.91 ± 1.98	4 (0–5)/221.76 ^a^			

SD: standard deviation; ES: effect size; CI: confidence interval. ^x^ Kruskal–Wallis test; ^y^ Mann–Whitney U test. ^a–c^ Different superscript letters within a variable indicate statistically significant differences between groups in post hoc pairwise comparisons (*p* < 0.05); groups sharing the same letter do not differ significantly.

**Table 6:** Comparison of awareness scores according to demographic characteristics.

	Mean ± SD	Median (Minimum–Maximum)/Mean Rank	Test Statistic	*p*	ES [95% CI]
Age					
>80	27 ± 2.26	28 (21–29)/290.29 ^ab^	16.072	0.001 ^x^	0.026 [0.000–0.060]
18–30	30.27 ± 4.33	30 (20–35)/381.07 ^a^			
31–50	26.99 ± 2.93	28 (19–35)/268.42 ^b^			
51–80	26.31 ± 3.16	27 (14–35)/242.37 ^b^			
Sex					
Male	26.51 ± 3.18	27 (14–35)/251.63	−0.112	0.911 ^y^	0.010 [−0.168–0.188]
Female	26.61 ± 3.24	27 (17–35)/253.1			
Duration of Diabetes (years)					
>16	26.28 ± 3.01	27 (17–35)/236.15	2.252	0.522 ^x^	−0.002 [−0.006–0.003]
1–5	26.43 ± 3.34	27 (14–35)/248.97			
11–15	26.71 ± 3.24	28 (21–35)/259.15			
6–10	26.83 ± 3.26	28 (19–35)/260.31			
Education Level					
Primary	25.7 ± 2.83	26 (20–35)/213.46 ^a^	45.447	<0.001 ^x^	0.083 [0.033–0.137]
Illiterate	26.83 ± 4.18	27 (19–35)/255.33 ^abc^			
Literate without formal schooling	25.51 ± 4.02	26 (17–35)/212.26 ^ab^			
Secondary	27.05 ± 2.92	28 (14–35)/276.49 ^bc^			
Higher education	28.5 ± 3.14	28 (21–35)/331.61 ^c^			
How often do you visit a dentist?					
Every 2 months	25.5 ± 3.54	25.5 (23–28)/209 ^ab^	27.439	<0.001 ^x^	0.049 [0.012–0.093]
Every 6 months	27.45 ± 3.08	28 (14–35)/292.84 ^a^			
Only for emergencies/when necessary	25.85 ± 3.08	26 (17–35)/218.88 ^b^			
Once a year	26.98 ± 3.27	28 (20–35)/271.76 ^a^			
How often do you brush your teeth?					
Once a day	25.98 ± 3.06	26 (21–35)/223.54 ^a^	28.374	<0.001 ^x^	0.051 [0.014–0.096]
More than twice a day	26.73 ± 2.84	27 (17–35)/257.89 ^ab^			
Twice a day	27.26 ± 3.28	28 (14–35)/287.65 ^b^			
Never	24.77 ± 3.81	24 (21–35)/152.96 ^a^			
Do you know about root canal infections and their symptoms?					
Yes	29.31 ± 2.76	28 (24–35)/370.04	−7.337	<0.001 ^y^	0.692 [0.437–0.946]
No	26.12 ± 3.06	27 (14–35)/233.23			
Have you ever had a tooth extracted due to a non-healing infection at the root canal?					
Yes	27.1 ± 3.31	28 (20–35)/274.51 ^a^	16.458	<0.001 ^x^	0.029 [0.003–0.066]
No idea	25.7 ± 2.88	26 (21–34)/208.04 ^b^			
No	26.7 ± 3.24	28 (14–35)/261.88 ^a^			

SD: standard deviation; ES: effect size; CI: confidence interval. ^x^ Kruskal–Wallis test; ^y^ Mann–Whitney U test. ^a–c^ Different superscript letters within a variable indicate statistically significant differences between groups in post hoc pairwise comparisons (*p* < 0.05); groups sharing the same letter do not differ significantly.

**Table 7:** Multiple linear regression analysis of factors associated with awareness score.

Variable	B (95% CI)	SE	β	t	*p*	Zero-Order	Partial	Part	VIF
Constant	27.003 (23.893 to 30.114)	1.566	—	17.245	<0.001	—	—	—	—
Age (years)									
>80	3.137 (−3.876 to 10.149)	3.530	0.085	0.889	0.377	0.041	0.093	0.079	1.173
18–30	0.536 (−2.538 to 3.609)	1.547	0.037	0.346	0.730	0.177	0.036	0.031	1.489
31–50	0.850 (−0.824 to 2.523)	0.843	0.104	1.008	0.316	0.175	0.105	0.089	1.369
51–80	Reference	—	—	—	—	—	—	—	—
Sex									
Male	−0.723 (−2.166 to 0.719)	0.726	−0.102	−0.996	0.322	−0.096	−0.104	−0.088	1.345
Female	Reference	—	—	—	—	—	—	—	—
Duration of diabetes (years)									
>16 years	−0.108 (−2.003 to 1.787)	0.954	−0.013	−0.113	0.910	−0.040	−0.012	−0.010	1.569
1–5 years	−0.013 (−1.863 to 1.836)	0.931	−0.002	−0.014	0.989	−0.050	−0.001	−0.001	1.791
11–15 years	1.360 (−0.574 to 3.293)	0.973	0.160	1.397	0.166	0.021	0.145	0.124	1.684
6–10 years	Reference	—	—	—	—	—	—	—	—
Education level									
Primary education	−2.630 (−4.903 to −0.357)	1.144	−0.364	−2.299	0.024	−0.265	−0.234	−0.203	3.210
Illiterate	−0.128 (−5.424 to 5.167)	2.666	−0.005	−0.048	0.962	0.098	−0.005	−0.004	1.325
Literate without formal schooling	−5.304 (−8.836 to −1.771)	1.778	−0.344	−2.982	0.004	−0.228	−0.298	−0.264	1.703
Secondary education	−1.732 (−3.683 to 0.218)	0.982	−0.236	−1.765	0.081	0.076	−0.182	−0.156	2.293
Higher education	Reference	—	—	—	—	—	—	—	—
How often do you visit a dentist?									
Every 2 months	−0.423 (−7.369 to 6.523)	3.497	−0.011	−0.121	0.904	−0.095	−0.013	−0.011	1.151
Every 6 months	1.181 (−0.795 to 3.157)	0.995	0.162	1.188	0.238	0.203	0.124	0.105	2.380
Only for emergencies/when necessary	0.847 (−1.124 to 2.818)	0.992	0.119	0.854	0.395	−0.153	0.089	0.076	2.471
Once a year	Reference	—	—	—	—	—	—	—	—
Do you know about root canal infections and their symptoms?									
Yes	1.547 (−0.437 to 3.531)	0.999	0.174	1.549	0.125	0.350	0.160	0.137	1.606
No	Reference	—	—	—	—	—	—	—	—
Have you ever had a tooth extracted due to a non-healing infection at the root canal?									
Yes	0.598 (−1.148 to 2.343)	0.879	0.075	0.680	0.498	0.112	0.071	0.060	1.561
No idea	−0.227 (−1.902 to 1.448)	0.843	−0.028	−0.269	0.788	−0.145	−0.028	−0.024	1.337
No	Reference	—	—	—	—	—	—	—	—

Model statistics: *F* = 2.162; *p* = 0.010; *R*^2^ = 28.88%; adjusted *R*^2^ = 15.55%; Durbin–Watson = 1.752. Note: B, unstandardized regression coefficient; β, standardized regression coefficient; SE, standard error; CI, confidence interval; VIF, variance inflation factor. Toothbrushing frequency was excluded from the multivariable model because its VIF exceeded 10, indicating problematic multicollinearity. —: not applicable.

**Table 8:** Multiple linear regression analysis of factors associated with knowledge score.

Variable	B (95% CI)	SE	β	t	*p*	Zero-Order	Partial	Part	VIF
Constant	4.199 (2.677 to 5.720)	0.766	—	5.481	<0.001	—	—	—	—
Age (years)									
>80	3.134 (−0.297 to 6.564)	1.727	0.161	1.814	0.073	0.108	0.187	0.149	1.173
18–30	−0.167 (−1.671 to 1.337)	0.757	−0.022	−0.221	0.826	0.133	−0.023	−0.018	1.489
31–50	0.688 (−0.131 to 1.507)	0.412	0.160	1.669	0.099	0.301	0.172	0.137	1.369
51–80	Reference	—	—	—	—	—	—	—	—
Sex									
Male	−0.468 (−1.174 to 0.238)	0.355	−0.125	−1.317	0.191	−0.100	−0.137	−0.108	1.345
Female	Reference	—	—	—	—	—	—	—	—
Duration of diabetes (years)									
>16 years	−0.200 (−1.127 to 0.727)	0.467	−0.044	−0.429	0.669	−0.038	−0.045	−0.035	1.569
1–5 years	−0.164 (−1.069 to 0.741)	0.456	−0.039	−0.359	0.720	0.050	−0.038	−0.029	1.791
11–15 years	−0.168 (−1.114 to 0.778)	0.476	−0.038	−0.353	0.725	−0.120	−0.037	−0.029	1.684
6–10 years	Reference	—	—	—	—	—	—	—	—
Education level									
Primary education	−1.962 (−3.073 to −0.850)	0.560	−0.515	−3.504	0.001	−0.423	−0.345	−0.288	3.210
Illiterate	−0.022 (−2.613 to 2.568)	1.304	−0.002	−0.017	0.986	0.043	−0.002	−0.001	1.325
Literate without formal schooling	−1.951 (−3.679 to −0.223)	0.870	−0.240	−2.243	0.027	−0.163	−0.229	−0.184	1.703
Secondary education	−0.618 (−1.572 to 0.336)	0.480	−0.160	−1.286	0.202	0.230	−0.134	−0.106	2.293
Higher education	Reference	—	—	—	—	—	—	—	—
How often do you visit a dentist?									
Every 2 months	−1.826 (−5.224 to 1.572)	1.711	−0.094	−1.067	0.289	−0.152	−0.111	−0.088	1.151
Every 6 months	0.456 (−0.510 to 1.423)	0.487	0.119	0.938	0.351	0.352	0.098	0.077	2.380
Only for emergencies/when necessary	−0.422 (−1.386 to 0.543)	0.485	−0.112	−0.868	0.387	−0.315	−0.091	−0.071	2.471
Once a year	Reference	—	—	—	—	—	—	—	—
Do you know about root canal infections and their symptoms?									
Yes	0.004 (−0.967 to 0.974)	0.489	0.001	0.007	0.994	0.273	0.001	0.001	1.606
No	Reference	—	—	—	—	—	—	—	—
Have you ever had a tooth extracted due to a non-healing infection at the root canal?									
Yes	−0.171 (−1.024 to 0.683)	0.430	−0.041	−0.397	0.692	0.049	−0.042	−0.033	1.561
No idea	0.057 (−0.763 to 0.876)	0.413	0.013	0.137	0.891	−0.138	0.014	0.011	1.337
No	Reference	—	—	—	—	—	—	—	—

Model statistics: *F =* 3.383; *p* < 0.001; *R*^2^ = 38.70%; adjusted *R*^2^ = 27.30%; Durbin–Watson = 1.780. Note: B, unstandardized regression coefficient; β, standardized regression coefficient; SE, standard error; CI, confidence interval; VIF, variance inflation factor. Toothbrushing frequency was excluded from the multivariable model because its VIF exceeded 10, indicating problematic multicollinearity. —: not applicable.

## Data Availability

The data supporting the findings of this study are available from the corresponding author upon reasonable request. The data are not publicly available because they contain information obtained from human participants and are subject to ethical and privacy restrictions.

## Supplementary Materials

The following supporting information can be downloaded at https://www.mdpi.com/article/10.3390/healthcare14213619/s1, Questionnaire S1: Diabetic patients’ knowledge and awareness of the relationship between diabetes mellitus and apical periodontitis.
