# Supplementary material for: Diabetic Patients’ Awareness and Knowledge of the Association Between Apical Periodontitis and Diabetes Mellitus: A Cross-Sectional Study
Source: Healthcare (Basel). 2026 Oct 26;14(21):3619. doi: 10.3390/healthcare14213619 (PMC13762555; doi:10.3390/healthcare14213619)
Supplement: Supplementary file 1 [file healthcare-14-03619-s001.zip › healthcare-4602544-supplementary.pdf]

Questionnaire S1: Diabetic patients' knowledge and awareness of the relationship between diabetes mellitus and apical periodontitis.

Part I: Demographic Information

1. **Age:** 18–30 / 31–50 / 51–80 / >80
2. **Sex:** Female / Male
3. **Type of Diabetes Mellitus:** Type 1 / Type 2
4. **Duration of Diabetes (years):** 1–5 / 6–10 / 11–15 / >16
5. **Fasting Blood Glucose level (mg/dL):** 70–100 mg/dL / 100–125 mg/dL / >126 mg/dL /
6. **Medication:** No-medication, Oral antidiabetic drugs (OAD), Combination of OAD and Insulin, Insulin only.
7. **Education Level:** Illiterate, Literate without formal schooling, Primary education, Secondary education, Higher education.
8. **Occupation:** Worker, Self-employed, Retired, Housewife.
9. **Smoking:** Current smoker, never smoked, Former smoker.
10. **How often do you brush your teeth?** Once a day, Twice a day, More than twice a day, Never.
11. **How often do you visit a dentist?** Once a year, Every 2 months, Every 6 months, Only for emergency/when necessary
12. **Current Oral Health Problems:** Dry mouth (Xerostomia), Delayed wound healing in the mouth, Gingival bleeding during brushing, Oral ulcers/sores, Dental caries (tooth decay), No problems.
13. **Do you have knowledge about root canal infections and their symptoms?** Yes / No
14. **Have you ever had a tooth extracted due to a non-healing root canal infection?** Yes / No / No idea.

Part II: Questions Assessing Knowledge Level

(Yes, No, No idea)

1	Diabetes Mellitus increases the risk of apical periodontitis
2	Diabetes Mellitus makes it harder to treat apical periodontitis
3	The presence of a apical periodontitis can prevent Diabetes Mellitus from being controlled.
4	Apical periodontitis follows a more severe course in diabetic patients.
5	The control of your Diabetes Mellitus is important for the healing of a tooth with apical periodontitis.

Part III: Questions Assessing Awareness Level

(Likert Scale: 1: Strongly Disagree, 2: Disagree, 3: neutral, 4: Agree, 5: Strongly Agree)

1. Gingival bleeding and tooth sensitivity require urgent dental treatment, especially if you have DM.
2. In patients with diabetes, apical periodontitis can be more severe or more difficult to manage.
3. Individuals with diabetes should pay closer attention to their oral health and have regular dental check-ups.
4. It is important to inform your dentist about your DM and the medications you use for a better dental treatment outcome.
5. Lifestyle, diet, and exercise changes can help manage DM, thereby reducing the risk of oral infections such as apical periodontitis.
6. Diabetic patients are more susceptible to dental infections, including apical periodontitis, than non-diabetic individuals.
7. High bacterial loads in the mouth of diabetic patients can make it more challenging to regulate blood sugar levels.
8. Certain diabetes medications may have side effects that affect oral health, such as dry mouth or delayed healing
